# Nutritional, Textural, and Sensory Attributes of Protein Bars Formulated with Mycoproteins

**DOI:** 10.3390/foods13050671

**Published:** 2024-02-23

**Authors:** Xiao-Yan You, Yue Ding, Qing-Yun Bu, Qin-Hong Wang, Guo-Ping Zhao

**Affiliations:** 1 Henan Engineering Research Center of Food Microbiology, College of Food and Bioengineering, Henan University of Science and Technology, Luoyang 471023, China; xiaoyanyou@haust.edu.cn (X.-Y.Y.); dingyue@haust.edu.cn (Y.D.); buqy@tib.cas.cn (Q.-Y.B.); 2 National Center of Technology Innovation for Synthetic Biology, Tianjin Institute of Industrial Biotechnology, Chinese Academy of Sciences, Tianjin 300308, China; 3 Haihe Laboratory of Synthetic Biology, Tianjin 300308, China; 4 CAS-Key Laboratory of Synthetic Biology, CAS Center for Excellence in Molecular Plant Sciences, Shanghai Institute of Plant Physiology and Ecology, Chinese Academy of Sciences, Shanghai 200032, China

**Keywords:** high-protein nutrition bars, mycoprotein, dietary fiber, sensory evaluation

## Abstract

Research accumulated over the past decades has shown that mycoprotein could serve as a healthy and safe alternative protein source, offering a viable substitute for animal- and plant-derived proteins. This study evaluated the impact of substituting whey protein with fungal-derived mycoprotein at different levels (10%, 20%, and 30%) on the quality of high-protein nutrition bars (HPNBs). It focused on nutritional content, textural changes over storage, and sensory properties. Initially, all bars displayed similar hardness, but storage time significantly affected textural properties. In the early storage period (0–5 days), hardness increased at a modest rate of 0.206 N/day to 0.403 N/day. This rate dramatically escalated from 1.13 N/day to 1.36 N/day after 5 days, indicating a substantial textural deterioration over time. Bars with lower mycoprotein levels (10%) exhibited slower hardening rates compared with those with higher substitution levels (20% and 30%), pointing to a correlation between mycoprotein content and increased bar hardness during storage. Protein digestibility was assessed through in vitro gastric and intestinal phases. Bars with no or low-to-medium levels of mycoprotein substitution (PB00, PB10, and PB20) showed significantly higher digestibility (40.3~43.8%) compared with those with the highest mycoprotein content (PB30, 32.9%). However, digestibility rates for all mycoprotein-enriched bars were lower than those observed for whey-protein-only bars (PB00, 84.5%), especially by the end of the intestinal digestion phase. The introduction of mycoprotein enriched the bars’ dietary fiber content and improved their odor, attributing a fresh mushroom-like smell. These findings suggest that modest levels of mycoprotein can enhance nutritional value and maintain sensory quality, although higher substitution levels adversely affect texture and protein digestibility. This study underscores the potential of mycoprotein as a functional ingredient in HPNBs, balancing nutritional enhancement with sensory acceptability, while also highlighting the challenges of textural deterioration and reduced protein digestibility at higher substitution levels.

## 1. Introduction

Ready-to-eat products have been increasingly attractive to consumers, among which high-protein nutrition bars (HPNBs) have become one of the most popular products owing to their convenience and health-oriented functions in muscle building, weight control and reduction, etc. [1,2,3,4]. HPNBs are intermedium-moisture food products with a water activity in the range of 0.6–0.8. This is low enough to inhibit the growth of most microorganisms and ensures a shelf life of between 6 and 12 months [3,4]. HPNBs normally contain 20~50% proteins, with carbohydrates, fat, sweeteners, and other ingredients added to create a pleasant flavor and taste and can effectively and quickly provide the energy needed by the body for performance [3]. In HPNB manufacturing, animal-derived proteins, such as milk whey protein and casein, are typically chosen as raw materials [5,6]. However, the production of animal proteins has low feed conversion efficiency and, therefore, is not environmentally and economically friendly [1,7,8,9]. Recently, the “green” trend, built on the idea of sustainability in the industry as well as in vegetarianism/vegan consumers’ choices, has inspired a great number of studies on the replacement of animal-derived proteins with nonanimal-derived dietary proteins that offer a good source and balance of amino acids.

Plant-derived proteins are the most commonly used ingredients to substitute animal-derived proteins. Soy, wheat gluten, and mushrooms are the main ingredients used [10]. While the bioavailability and amino acid profiles of certain plant-based proteins may resemble those of eggs, the presence of antinutrients like phytates, tannins, protease inhibitors, and saponins can impede the absorption of these proteins [11]. Furthermore, employing mechanical and thermal preprocessing techniques (such as roasting, dehulling, blanching, soaking, cooking, and sprouting) can mitigate antinutrients like protease inhibitors, although certain antinutrients remain resilient and cannot be entirely eliminated [12]. In light of these factors, there is an imperative to formulate healthful food products that encompass all essential amino acids, or at the very least, a majority of them, while being devoid of antinutrients that curtail their bioavailability.

Mycoprotein, derived from the fermentation of the filamentous fungus *Fusarium venenatum*, presents a nutritional profile superior to that of both animal- and plant-derived proteins. This includes a reduction in saturated fat content, an elevation in dietary fiber content, and a rich composition of essential amino acids [13]. In addition, mycoprotein has been deemed safe for consumption by the U.S. Food and Drug Administration (FDA) and has been legally available in more than 10 countries since 1985 [14,15]. As such, it possesses the potential to partially or completely supplant animal- and plant-derived protein sources [16,17]. While mycoprotein has primarily been introduced over the past few decades as a sustainable and healthful dietary protein in the development of meat alternatives [18,19], to the best of our knowledge, no research has hitherto explored its integration into high-protein nutrition bars (HPNBs). In the present study, mycoprotein obtained from *Fusarium venenatum* TB01 was introduced for the preparation of protein bars that were originally based only on milk whey protein isolate. Three different substitution levels (10%, 20%, and 30%) of mycoprotein were used. The nutritional compositions, digestion-related properties, as well as sensory profiles of the novel bars formulated with mycoproteins were investigated. The hardening behaviors of bars during storage were also compared. This study provides fundamental information for the future incorporation of mycoproteins into ready-to-eat high-protein nutrition bars.

## 2. Materials and Methods

### 2.1. Materials

Mycoprotein was provided by Tianjin Institute of Industrial Biotechnology (Tianjin, China), and its major nutrition profiles (Table S1) were determined by Hangzhou Yanqu Information Technology Co. Ltd. Milk whey protein isolate (WPI, containing 93% protein) was supplied by Hilmar Ingredients Inc. (Hilmar, CA, USA). The amino acid contents and qualities of mycoprotein are listed in Table S3. Cooked rice powder (containing 6.8% protein, 0.6% fat, and 80.2% carbohydrate, according to the manufacturer), food-grade glycerol (>98%), liquid sorbitol (>75%), salt, and butter (0.6% protein, 82.9% fat, 0.6% carbohydrate, 10 mg Na) were all purchased from a local supermarket of food additives (Hongjin, Luoyang, China). Pepsin (P6322, 3000 U/mg), chymotrypsin (C804761, 800 U/mg), α-amylase (A834632, 50 U/mg), and lipase (L874999, 90 U/mg) used in in vitro digestion experiments were purchased from Macklin (Shanghai, China). Trypsin (S10034, 250 U/mg) was obtained from Yuanye (Shanghai, China). All other chemicals used in the study were purchased from Macklin (Shanghai, China).

### 2.2. Preparation of Protein Bars

Cold processing method was used to prepare the HPNBs [3,6]. The ingredients, including whey protein isolate, mycoprotein, cooked rice powder, food-grade glycerol, sorbitol, and 20% (*w*/*w*) salt solution, were combined at room temperature with a KitchenAid mixer on speed 3 for 5 min. Then melted butter was added. The mixing was continued for 2 min to form uniform dough. A total of 30 g of dough was packed into a rectangular bar frame with 6.7 cm length, 3.4 cm width, and 2.2 cm height and was leveled with a spatula. For each formulation of the protein bars, we prepared a batch of samples (about 40 bars) for testing purposes. The prepared bars were placed in polyethylene bags and stored at 25 °C for 15 days.

The ingredients used for protein bars are given in Table 1. Model bars with 10%, 20%, and 30% whey protein were substituted by mycoprotein, which was added at a level to keep the crude protein content of all the model bars to be approximately 36 g per 100 g bar. Cooked rice powder was incorporated so that the total content of dry ingredients was the same for all the formulations (i.e., 58 g per 100 g bar). Other ingredients, including the food-grade glycerol, sorbitol, NaCl solution, and butter, were used at the same level.

Model protein bars with 10%, 20%, and 30% whey protein substituted by mycoprotein were labeled as PB10, PB20, and PB30, respectively. The bars that only used whey protein isolate as protein source without any incorporation of mycoprotein were referred to as PB00.

### 2.3. Proximate Composition Analysis

The proximate composition of bars was determined according to AOAC official methods: 992.23 (crude protein), 920.39 (crude fat), 2009.01 (dietary fiber), and 923.03 (ash). The moisture content of sample was obtained by drying 5 g sample in an oven at 120 °C for 24 h, and the reduction in weight was taken as the moisture content. The crude carbohydrate content was determined according to the following formula [20]:

Carbohydrate (%)=100−(% protein+% fat+% dietary fiber+% ash+% moisture)

and the caloric values for each category were calculated as follows:

Energy value (Kcal/100 g)=(4×% protein)+(9×% fat)+(4×% carbohydrate)

### 2.4. Change in Bar Hardness

The analysis of bar hardness was conducted using a TA.XT Plus Texture Analyzer (Stable Micro Systems, Godalming, UK) following the study of Dan Zhu and Labuza (2010), with slight modifications [21]. The test model (within software Texture Expert V 2.64) was punctured with a cylinder probe (P/2 stainless steel, 2 mm in diameter). During the testing, the speed of pretest, test, and post-test was set to be 2, 1, and 2 mm/s, respectively, and the target distance was 10 mm. A force–distance curve was obtained during each test, in which the maximum positive force was referred to as the hardness of the bar. The bars were measured for their hardness when they were freshly prepared (day 0) and also on the 1st, 3rd, 5th, 10th, and 15th day of storage. The hardening rate (i.e., the average speed of the increase in hardness over a time period) at the early and late stages of storage was also calculated. The formula was as follows,

$$Hardening\;rate\;(N/day)=\frac{{Hardness}_{2}-{Hardness}_{1}}{Time\;period}$$

in which ${Hardness}_{1}$ and ${Hardness}_{2}$ are the hardness of the sample bar measured at the beginning and the end of a time period.

### 2.5. In Vitro Protein Digestibility Test

In vitro protein digestion of bar samples was conducted following the INFOGEST protocol, with some modifications [22]. The freshly made bar sample (5 g) was suspended in 15 mL simulated gastric fluid (SGF, pH 2.0, 6.9 mM KCl, 0.9 mM KH_2_PO_4_, 35 mM NaCl). The gastric phase was started by adding 30 mg pepsin into the sample-SGF suspension. The digestion took place at 37 °C for 2 h with shaking at 200 rpm, during which the pH was kept at 2.0 using addition of 3 M HCl. Then, the pH of the suspension was adjusted to 7.0 with 3 M NaOH to stop the gastric phase. Subsequently, 15 mL simulated intestinal fluid (SIF, pH 7.0, 6.8 mM KCl, 0.8 mM KH_2_PO_4_, 50 mM NaCl) was added. Then, the intestinal phase was started with addition of a mixture of enzymes (containing 288 mg trypsin, 24 mg chymotrypsin, 400 mg α-amylase, and 400 mg lipase), and the process was continued at 37 °C for 2 h with pH kept at 7.0 using 3 M NaOH. The digestion was stopped by the addition of 10 mL of 10% trichloroacetic acid (TCA).

At the end of both gastric and intestinal phases, the samples were centrifuged at 20 °C, 12,000× *g* for 10 min. The nitrogen content in the supernatant was determined by multi N/C 2100 analyzer using Dumas (combustion) method and converted to crude protein content by multiplying a factor of 6.25. All determinations were performed in triplicate. In vitro protein digestibility of bars was calculated according to the following formulas,

$$Digestibility\;(\%)=\frac{{Crude\;protein}_{supernatant}}{Total\;crude\;protein}\times 100$$

### 2.6. Microstructure

The microstructure of protein bars was examined at 0, 7, and 14 days using confocal scanning laser microscopy (CSLM) equipped with an Ar/Kr laser [13]. A thin slice was cut from the middle of the bar samples and stained with 0.02% fluorescein isothiocyanate (FITC) solution (0.02 g FITC in 100 mL absolute acetone) to label protein. The staining was allowed for 1 min.

Argon laser was used to excite the FITC at a wavelength of 488 nm. The fluorescence emitted from the sample was captured at a wavelength of 512 nm. The micrographs were acquired and analyzed by ZEN 2012 software.

### 2.7. Sensory Evaluation

The protein bars used in sensory evaluation were freshly prepared and formed into a cuboid (5 cm × 3.4 cm × 2.2 cm). For each evaluation, the bar sample was randomly assigned with a two-digit code and was provided to the panelists on a plastic plate.

The sensory properties of protein bar samples were assessed by 20 participants in terms of appearance, color, odor, taste, aftertaste, texture, and overall acceptability. The participants were all first-year or second-year postgraduates from Henan University of Science and Technology aged between 23 and 25 years old. Before the assessment, the panelists were briefly trained in the definition of these sensory attributes (Table S2). They were then asked to rate each bar according to their degree of desirability for these sensory attributes, using a 10-point hedonic scale in which 1 means “dislike extremely” and 10 means “like extremely” [23,24]. Between the evaluations, the panelists were provided with water to rinse their mouths.

### 2.8. Statistical Analysis

All experiments were conducted in triplicate. The mean values and standard deviations were analyzed using Excel 365, and the significance between means was determined using one-way ANOVA at a significance level of *p* < 0.05, followed by Duncan’s multiple comparison tests.

## 3. Results and Discussions

### 3.1. Proximate Composition Analysis of HPNBs

The compositions of protein bars, varying in the extent of whey protein isolate (WPI) replacement with mycoprotein (MP), were subject to approximate analysis and are presented in Table 2. Given the substantial variance in protein content between mycoprotein (35.6%, *w*/*w*) and WPI (93%, *w*/*w*), the incorporation of mycoprotein was guided by the formulations outlined in Table 1. This approach aimed to maintain a relatively consistent crude protein content across bars within each category. To ensure the uniformity of the dough, cooked rice powder was added in a quantity that preserved the total dry ingredients at 58 g per 100 g bar.

From the data presented in Table 2, it is evident that all of our model bars exhibited comparable protein content, ranging from 36.06% to 37.63%. This content surpasses that found in commercial protein bars, which typically contain 15 to 30 g of protein per 100 g [1,25]. With an increasing incorporation of mycoprotein, there was a notable rise in fat and dietary fiber content. This increase can be attributed to mycoprotein’s composition, notably its substantial crude fat content (14.7%) and, particularly, its dietary fiber content (42.9%, Table S1). The well-established recognition of the health benefits associated with dietary fiber consumption, including the reduced risk of developing conditions such as hypertension, diabetes, obesity, and certain gastrointestinal disorders, is worth noting [26]. Remarkably, our model protein bars—PB10, PB20, and PB30—where 10%, 20%, and 30% of whey protein isolate (WPI) was replaced by mycoprotein, offered approximately 13.2 g, 24.6 g, and 36.7 g of dietary fiber per 1000 kcal, respectively. This substantial dietary fiber content aligns with the recommended daily intake of dietary fiber (approximately 14 g/1000 kcal) and positions these bars as wholesome, fiber-rich options for health-conscious consumers [26,27].

**Figure 1:**
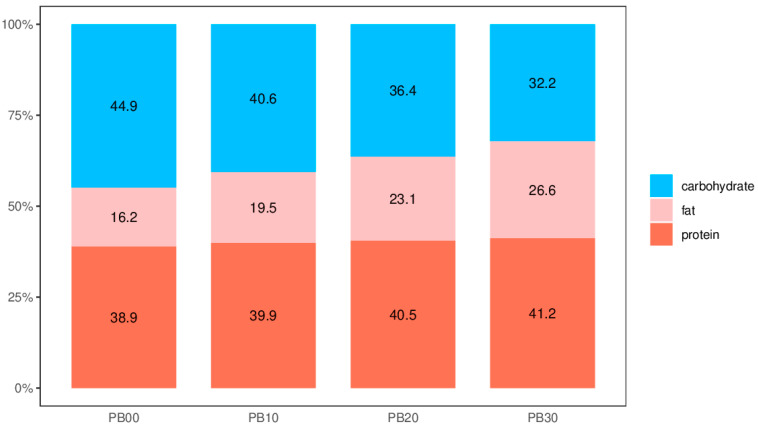
The distribution of calories supplied by macronutrients in model high-protein nutrition bars of different formulations. PB10, PB20, and PB30 are model protein bars with whey protein isolate replaced by mycoprotein at 10%, 20%, and 30%, respectively. PB00 represents control bars formulated exclusively with whey protein isolate without mycoprotein substitution.

Regarding energy content, the protein bars in our investigation exhibited a range of approximately 351.01 to 386.65 kcal per 100 g. This aligns with the energy content observed in both the literature’s most studied protein bars (330 to 410 kcal/100 g) and commercially available options (340 to 430 kcal/100 g), contingent on brand and flavor preferences [1,2,28]. Caloric distribution analysis of our model protein bars (Figure 1) unveiled energy contributions from carbohydrates, fat, and protein within ranges of 32.2% to 44.9%, 16.2% to 26.6%, and 38.9% to 41.2%, respectively. It is worth noting that certain distributions deviated from the acceptable macronutrient distribution range (AMDR), set at 45% to 65% for carbohydrates, 20% to 35% for fat, and 10% to 35% for protein, as recommended by the Food and Nutrition Board of the Institute of Medicine (IOM) [29,30], reflecting the protein-rich nature of high-protein and high-fiber nutrition bars (HPNBs). Furthermore, the moisture content of bars containing added mycoprotein in our current study averaged around 10%, consistent with typical levels found in most commercial HPNBs [3]. Proximate analysis results indicated that our model protein bars, enriched with mycoprotein, constitute medium-moisture food products boasting substantial protein and dietary fiber content while maintaining a modest fat content.

### 3.2. Change in Bar Hardness

The hardness of HPNBs typically increases during storage, which is harmful to the quality of this kind of food product. The shelf life of protein bars is often limited by this hardening effect [3,31].

**Figure 2:**
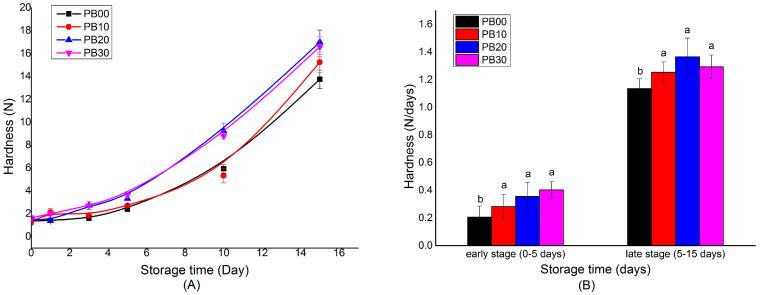
The hardening process of model high-protein nutrition bars during storage at 25 °C for 15 days (**A**) and the calculated average hardening rate (**B**). PB10, PB20, and PB30 are model protein bars with whey protein isolate replaced by mycoprotein at 10%, 20%, and 30%, respectively. PB00 represents control bars formulated exclusively with whey protein isolate without mycoprotein substitution. Data displayed are mean values ± standard deviation (*n* = 3). The mean values are significantly different (*p* < 0.05) if they do not share a common superscript lowercase letter.

The hardness of bar samples was measured on days 0, 1, 3, 5, 10, and 15 when stored at 25 °C for 15 days. The changes in the hardness are illustrated in Figure 2. It was seen that the model protein bars hardened at different rates depending on the stages during storage. The bar samples made from different formulations all displayed a similar hardness when they were freshly prepared (day 0). In the early stage of storage (0~5 days), the hardening of bars proceeded at a relatively low level, varying from 0.206 N/day to 0.403 N/day (Figure 2B), while it accelerated at a much higher speed from the fifth day during storage (ranging from 1.13 N/day to 1.36 N/day), which was roughly 3~5 times that of the early stage.

Moreover, the hardening of bars was also influenced by their formulation. Generally speaking, the bar samples hardened more rapidly with an increasing amount of mycoprotein being incorporated. The protein bars without the addition of mycoprotein (PB00) and with mycoprotein added at a low level of 10% (PB10) hardened at a rate of 0.206 N/day and 0.282 N/day, respectively, in the early stage. In contrast, the rate was 0.356 N/day and 0.403 N/day for bars with more substitution (PB20 and PB30). The difference became much more distinct in the late stage of storage (Figure 2A). From the fifth day, the hardness of PB20 and PB30 was dramatically higher than that of PB00 and PB10.

The hardening of HPNBs during storage is the consequence of the complex physiochemical reactions taking place during the storage period. It was proposed that a multiple-protein system could delay the hardening of protein bars to some extent [3,23]. Unfortunately, this was not the case for our samples. The incorporation of mycoprotein generated a higher hardening rate compared with bars only made from a single type of protein. In the current study, it was observed that the overall textural properties of bars with and without the addition of mycoprotein diverged during storage. These differences were supposed to be related to the structural characteristics of proteins as well as their interactions with the environment (such as moisture and other macronutrients) [3]. Our results suggested that whether a multiple-protein system could have a delaying effect in terms of bar hardening depends on the intrinsic properties of the proteins used as well as their interactions with other ingredients.

### 3.3. In Vitro Protein Digestibility Test

**Figure 3:**
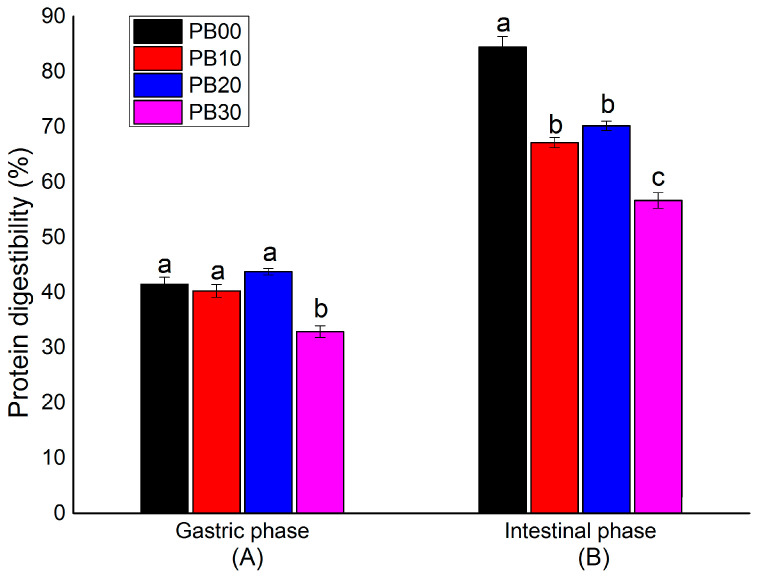
In vitro protein digestibility (%) by the end of the (**A**) gastric phase and (**B**) intestinal phase. The mean values are significantly different (*p* < 0.05) if they do not share a common superscript lowercase letter. PB10, PB20, and PB30 are model protein bars with whey protein isolate replaced by mycoprotein at 10%, 20%, and 30%, respectively. PB00 represents control bars formulated exclusively with whey protein isolate without mycoprotein substitution. Data displayed are mean values ± standard deviation (*n* = 3). The mean values are significantly different (*p* < 0.05) if they do not share a common superscript lowercase letter.

The percentages of protein digested by the end of both gastric and intestinal phases were measured in the current in vitro digestibility test. This offered information on the protein accessibility for adsorption (Figure 3). The protein digestibility of bars during the gastric phase was similar unless the substitution level was too high (Figure 3A). The bars without mycoprotein (PB00) and with mycoprotein substituted at a low or medium level (PB10 and PB20) achieved a protein digestibility varying from 40.3~43.8%, which was significantly higher than the bars with the highest substitution level (PB30, protein digestibility at 32.9%). In comparison, it was seen from Figure 3B that by the end of the intestinal phase, a total amount of 56.7~67.2% protein had been digested for bar samples with different amounts of mycoprotein added (PB10, PB20, and PB30), which was remarkably lower than the bars based only on whey protein isolate (PB00, protein digestibility at 84.5%). Among PB10, PB20, and PB30, the protein digestibility of PB30 was significantly lower than that of PB10 and PB20, whereas there was no significant difference between PB10 and PB20.

**Figure 4:**
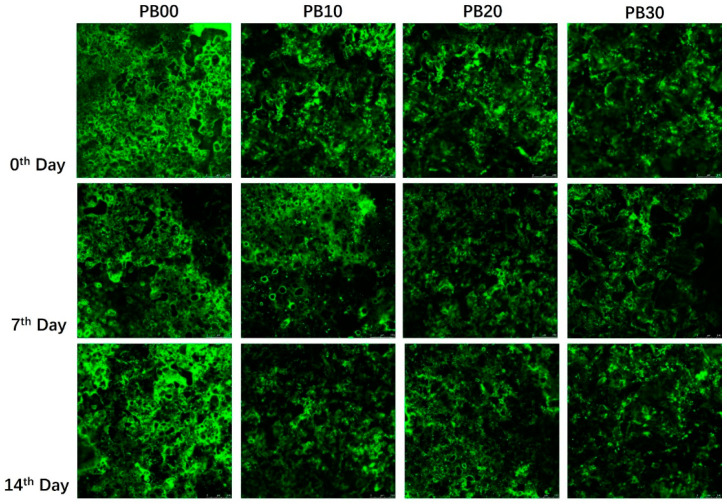
Confocal micrographs of the microstructure of protein bars with different formulations. PB10, PB20, and PB30 are model protein bars with whey protein isolate replaced by mycoprotein at 10%, 20%, and 30%, respectively. PB00 represents control bars formulated exclusively with whey protein isolate without mycoprotein substitution.

The observed differences in protein digestibility among the samples during the gastric and intestinal phases are believed to be associated with the structural characteristics of the mycoprotein and the bars produced from it. The mycoprotein utilized in this study is notably rich in dietary fiber (42.9%), which contributes to its slightly porous structure. Furthermore, bars incorporating mycoprotein exhibited a distinctly loose texture, as evidenced by the microstructural analysis presented in Figure 4. The micrographs reveal a significant variation in the microstructural organization between the protein bars with mycoprotein incorporation (PB10, PB20, and PB30) and those without (PB00). In the confocal micrographs, the proteins in PB00 appear brighter green, indicating a denser and more uniform protein network across different time points. Conversely, the bars with mycoprotein (PB10, PB20, and PB30) demonstrated a more loosely packed and heterogeneous protein network with a gradient in concentration. Hence, while the bars based solely on whey protein (PB00) were easier to digest, the inclusion of low-to-medium levels of mycoprotein (in PB10 and PB20) resulted in a relatively loose structure. This structural characteristic facilitated enzyme access, allowing these samples to exhibit comparable protein digestibility during the gastric phase. However, bars with a large amount of mycoprotein (PB30) were shown to have a significantly lower rate of protein digestion in the intestinal phase, probably due to their dietary-fiber-rich attribute and, typically, the hyphal structure of mycoprotein [8,32]. Fungal cell walls (FCWs) are composed of matrix components that are embedded and linked to scaffolds of fibrous load-bearing polysaccharides. Intracellular nutrients from the fungal cell must be accessible to enzymes to be digested and then absorbed. A growing body of studies has consistently shown that fibrous cell walls control (limit or prevent) the release of nutrients from food matrices [33]. Colosimo et al. (2020) found that the porosity/permeability of enzyme diffusion through cell walls is the main factor responsible for the hydrolysis and bioaccessibility of mycoprotein [32]. The lower sample concentration (10 wt % vs. 25 wt %) released a higher proportion of protein at the simulated digestion endpoints following the use of Driselase^TM^, suggesting that a high concentration of mycoprotein hyphae will reduce the protein released from the mycoprotein samples [34]. It was most likely this characteristic of mycoprotein that was responsible for the much lower protein digestibility of PB10, PB20, and PB30 by the end of the intestinal phase (Figure 3).

These results from in vitro protein digestion indicated that the introduction of mycoprotein to high-protein nutrition bars could have a negative effect on protein accessibility. The controlled bioaccessibility of digestive enzymes to gain access to and hydrolyze nutrients not only limits the protein release of mycoprotein but also limits energy availability from diet. Therefore, this will be a possible mechanism underlying the health effects of mycoprotein by promoting satiety and attenuating postprandial glycemia. However, due to the known limitations of in vitro digestion experiments (such as the lack of simulation of the dynamics of the digestion process or the physiological interactions with the body) [22], how protein bioaccessibility could be affected remains to be solved by in vivo digestion.

### 3.4. Sensory Evaluation

**Figure 5:**
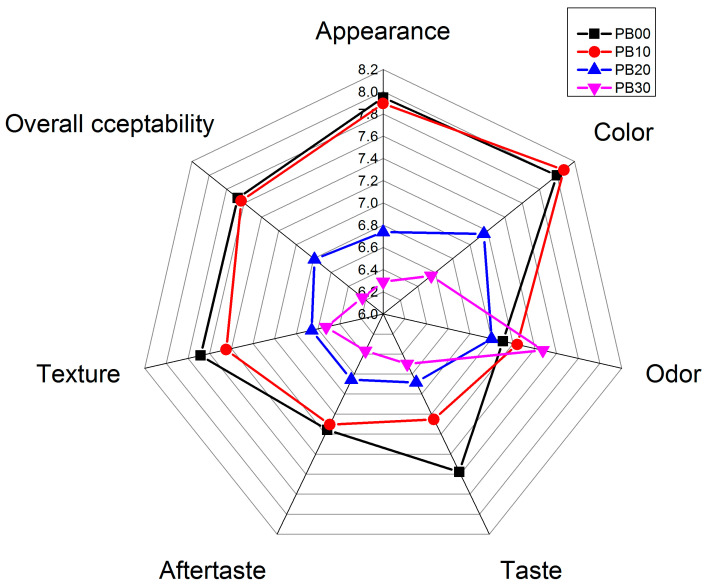
Sensory properties of model high-protein nutrition bars. PB10, PB20, and PB30 are model protein bars with whey protein isolate replaced by mycoprotein at 10%, 20%, and 30%, respectively. PB00 represents control bars formulated exclusively with whey protein isolate without mycoprotein substitution.

A sensory evaluation involving 20 Chinese postgraduates aged 23 to 25 was conducted to assess the freshly prepared bars. The outcomes, presented in Figure 5 and Table 3, revealed the preferences of young consumers toward the bar samples. Notably, PB00 and PB10 garnered similar ratings across various sensory attributes, such as appearance, color, texture, and overall acceptability. These ratings were significantly higher compared with PB20 and PB30 (bars with increased mycoprotein content), except for odor and aftertaste. Intriguingly, the bars with the highest substitution level (PB30) were noticeable in terms of odor, possibly due to the alluring, fresh mushroom-like aroma resonating well with the young populace.

The introduction of mycoprotein did enhance the bars’ taste; the highest score was awarded to PB00, crafted solely from milk whey protein isolate. Panelists noted that while mycoprotein possessed an appealing fragrance, its taste was not reminiscent of mushrooms but rather somewhat resembled undercooked rice. This likely accounted for the comparatively lesser preference for bars with added mycoprotein and their associated aftertaste. In addition, panelists observed a nonuniform milky color as well as a rougher surface in mycoprotein-containing bars, which became particularly obvious in the ones with higher levels of mycoproteins (PB20 and PB30). A slightly granular mouthfeel was also detected in PB20 and PB30, akin to whole wheat or whole oat products, potentially linked to mycoprotein’s dietary-fiber-rich nature. As for the texture of the bars, it was reported by the panelists that the incorporation of mycoprotein had some negative effects. Bars with the addition of mycoproteins were found to be much more rigid and fragile, while the bars formulated only using whey protein were reported to be firmer and more elastic. This greatly increased rigidness and fragility were not found to be pleasant and could consequently elucidate the subdued scores accorded by most young participants to mycoprotein-incorporated bars. The findings indicated that moderate substitution levels (around 10%) of whey protein with mycoprotein minimally impacted consumer acceptance of sensory attributes. To enhance the palatability of mycoprotein-enriched bars, adjustments to textural and taste properties would be advisable.

## 4. Conclusions

As a high-quality alternative protein, mycoprotein is typically used by the food industry to produce meat analogs; however, this is relatively limited. The current work aimed to explore the possibility of incorporating mycoprotein in making whey-protein-based high-protein nutrition bars (HPNBs). The whey protein ingredient was partially substituted by mycoprotein at three different levels (10%, 20%, and 30%), and the effects of mycoprotein incorporation on the main nutritional, textural, and sensory properties of bars were assessed. Our findings highlight that all bar formulations maintained similar hardness when freshly prepared, with significant variations in hardening rates observed during storage. Notably, bars with higher mycoprotein content exhibited a dramatic increase in hardness after the fifth day, suggesting a notable influence of mycoprotein on the textural stability of HPNBs over time. The digestibility of the protein during the gastric phase remained relatively consistent across bars with low-to-medium levels of mycoprotein substitution, indicating that moderate inclusion does not adversely affect protein bioaccessibility. However, the highest substitution level resulted in significantly lower protein digestibility, emphasizing the need for a balanced approach in mycoprotein incorporation. The addition of mycoprotein not only enriched the dietary fiber content but also improved the sensory attributes of the bars, attributed to their dietary-fiber-rich properties and fresh mushroom-like smell. Bars with modest mycoprotein substitution achieved comparable overall acceptability to those containing only whey protein, underscoring the potential of mycoprotein as a viable ingredient in HPNBs.

Our study provides foundational insights into the feasibility of integrating mycoprotein into HPNBs and potentially other health-oriented food products. Future research is crucial for refining the control over mycoprotein’s protein release kinetics and maximizing its health benefits. Collaborative efforts between academia and the food industry are imperative to address storage-related challenges and develop optimized formulations. Such endeavors will not only enhance our understanding of mycoprotein’s benefits but also facilitate its successful market integration, appealing to a broad consumer base seeking nutritious and convenient food options.

## Figures and Tables

**Table 1:** The ingredient used for protein bars.

Ingredient (g/100 g)	PB00	PB10	PB20	PB30
Whey protein isolate	40	36	32	28
Mycoprotein	-	10	20	30
Cooked rice powder	18	12	6	-
Glycerol	16	16	16	16
Liquid sorbitol	20	20	20	20
20% (*w*/*w*) salt solution	1	1	1	1
Butter	5	5	5	5

Note: PB10, PB20, and PB30 are model protein bars with whey protein isolate replaced by mycoprotein at 10%, 20%, and 30%, respectively. PB00 represents control bars formulated exclusively with whey protein isolate without mycoprotein substitution.

**Table 2:** The proximate composition of model high-protein nutrition bars with different formulations.

Nutritional Composition	Protein (%)	Fat (%)	Carbohydrate (%)	Dietary Fiber (%)	Ash (%)	Moisture (%)	Energy (kcal/100 g)
PB00	37.63 ± 0.43 ^a^	6.89 ± 0.12 ^c^	43.46 ± 0.88 ^a^	0.95 ± 0.12 ^a^	1.26 ± 0.08 ^a^	9.47 ± 0.24 ^c^	386.37 ± 6.32 ^a^
PB10	37.11 ± 0.26 ^a^	8.06 ± 0.31 ^b^	37.69 ± 1.21 ^b^	4.92 ± 0.56 ^b^	1.54 ± 0.12 ^bc^	10.21 ± 0.16 ^ab^	371.74 ± 8.67 ^b^
PB20	36.58 ± 0.35 ^b^	9.22 ± 0.24 ^a^	32.38 ± 0.67 ^c^	8.90 ± 0.64 ^c^	1.81 ± 0.20 ^c^	10.47 ± 0.08 ^a^	360.58 ± 6.24 ^c^
PB30	36.06 ± 0.42 ^c^	10.38 ± 0.89 ^a^	28.22 ± 1.21 ^d^	12.87 ± 1.10 ^d^	2.09 ± 0.06 ^c^	10.18 ± 0.15 ^b^	350.54 ± 10.53 ^c^

Note: PB10, PB20, and PB30 are model protein bars with whey protein isolate replaced by mycoprotein at 10%, 20%, and 30%, respectively. PB00 represents control bars formulated exclusively with whey protein isolate without mycoprotein substitution. Data displayed are mean values ± standard deviation (*n* = 3). The mean values are significantly different (*p* < 0.05) if they do not share a common superscript lowercase letter.

**Table 3:** The statistical analysis of different sensory criteria of model high-protein nutrition bars.

Attribute	PB00	PB10	PB20	PB30
Appearance	7.95 ± 1.03 ^a^	7.89 ± 1.10 ^a^	6.74 + 1.08 ^b^	6.29 ± 1.17 ^b^
Color	8.00 ± 0.94 ^a^	8.08 ± 0.95 ^a^	7.16 ± 1.05 ^b^	6.55 ± 1.26 ^b^
Odor	7.11 ± 1.66 ^ns^	7.24 ± 1.42 ^ns^	7.00 ± 0.88 ^ns^	7.47 ± 1.07 ^ns^
Taste	7.58 ± 1.43 ^a^	7.05 ± 1.35 ^a,b^	6.68 ± 1.34 ^b^	6.50 ± 1.40 ^b^
Aftertaste	7.16 ± 1.34 ^ns^	7.11 ± 1.56 ^ns^	6.66 ± 1.55 ^ns^	6.37 ± 1.57 ^ns^
Texture	7.68 ± 1.20 ^a^	7.45 ± 1.34 ^a,b^	6.66 ± 1.37 ^b^	6.53 ± 1.87 ^b^
Overall acceptability	7.67 ± 1.24 ^a^	7.63 ± 1.13 ^a,b^	6.79 ± 1.24 ^b^	6.24 ± 1.25 ^c^

Note: PB10, PB20, and PB30 are model protein bars with whey protein isolate replaced by mycoprotein at 10%, 20%, and 30%, respectively. PB00 represents control bars formulated exclusively with whey protein isolate without mycoprotein substitution. Data displayed are mean values ± standard deviation (*n* = 3). The mean values are significantly different (*p* < 0.05) if they do not share a common superscript lowercase letter. ns means not significant.

## Data Availability

The original contributions presented in the study are included in the article/supplementary material, further inquiries can be directed to the corresponding authors.

## Supplementary Materials

The following supporting information can be downloaded at: https://www.mdpi.com/article/10.3390/foods13050671/s1, Table S1: The major nutrition profiles of mycoprotein (per 100 g), Table S2: The definitions of sensory attributes, Table S3: Amino acids content comparison of mycoprotein and other proteins.
