# Supplementary material for: Nutritional, Textural, and Sensory Attributes of Protein Bars Formulated with Mycoproteins
Source: Foods. 2024 Feb 23;13(5):671. doi: 10.3390/foods13050671 (PMC10930427; doi:10.3390/foods13050671)
Supplement: Supplementary file 1 [file foods-13-00671-s001.zip › foods-2856527-supplementary.pdf]

Table S1. The major nutrition profiles of mycoprotein (per 100 g).

Items	Results
Proteins	35.6
Total fat	7.8
Ash content	4.6
Total dietary fiber	42.9

Table S2. The definitions of sensory attributes.

Attributes	Definitions
**Appearance**	the extent to which the smoothness and graininess of the bar surface is found to be appealing
**Color**	the uniformity of the color of the bar
**Odor**	the extent to which the smell of the bar is found to be pleasant
**Taste**	the extent to which the balance of the sweetness, saltiness, the milky and the mushroom-like flavor of the bar is found to be pleasant
**Aftertaste**	the extent of the pleasant taste left in the mouth after swallowing
**Texture**	the extent to which the rigidness, fragility, firmness and elasticity of the bar is found to be pleasant by the mouth
**Overall acceptance**	the extent to which the overall quality of the bar is found to be pleasant

Table S3. Amino acids content comparison of mycoprotein and other proteins.

Amino acids	MP	Soy*	Wheat*	Pea*	Corn*	Potato*	Egg*	Whey*	Milk*
**Essential amino acids （EAAs）**									
**Threonine**	2.20	2.3	1.8	2.5	1.8	4.1	2.0	5.4	3.5
**Methionine**	1.42	0.3	0.7	0.3	1.1	1.3	1.4	1.8	2.1
**Phenylalanine**	1.69	3.2	3.7	3.7	3.4	4.2	2.3	2.5	3.5
**Histidine**	0.91	1.5	1.4	1.6	1.1	1.4	0.9	1.4	1.9
**Lysine**	3.52	3.4	1.1	4.7	1.0	4.8	2.7	7.1	5.9
**Valine**	3.97	2.2	2.3	2.7	2.1	3.7	2.0	3.5	3.6
**Isoleucine**	2.04	1.9	2.0	2.3	1.7	3.1	1.6	3.8	2.9
**Leucine**	3.26	5.0	5.0	5.7	8.8	6.7	3.6	8.6	7.0
**∑ EAA**	**19.01**	**19.9**	**18.0**	**23.6**	**21.0**	**29.3**	**16.5**	**34.1**	**30.3**
**Non-essential amino acids （NEAAs）**									
**Serine**	1.95	3.4	3.5	3.6	2.9	3.4	3.3	4.0	4.0
**Glycine**	1.93	2.7	2.4	2.8	1.6	3.2	1.4	1.5	1.5
**Glutamic acid**	4.22	12.4	26.9	12.9	13.1	7.1	5.1	15.5	16.7
**Proline**	2.22	3.3	8.8	3.1	5.2	3.3	1.8	4.8	7.3
**Cysteine**	ND	0.2	0.7	0.2	0.3	0.3	0.4	0.8	0.2
**Alanine**	2.82	2.8	1.8	3.2	4.8	3.3	2.6	4.2	2.6
**Tyrosine**	1.53	2.2	2.4	2.6	2.7	3.8	1.8	2.4	3.8
**Arginine**	2.66	4.8	2.4	5.9	1.7	3.3	2.6	1.7	2.6
**Aspartate**	3.02	ND	ND	ND	ND	ND	ND	ND	ND
**∑ NEAA**	**17.33**	31.9	48.9	34.4	32.3	27.8	19.0	34.9	38.6
**∑ TAA（Total amino acids）**	**39.36**	**51.8**	**66.9**	**58.0**	**53.3**	**57.1**	**35.5**	**69.0**	**68.9**

Note: Values are presented in g per 100 g raw material. *Data obtained from Stefan H. M. Gorissen. *et.al*.
